# Identification of Immunity-Related Genes in *Ostrinia furnacalis* against Entomopathogenic Fungi by RNA-Seq Analysis

**DOI:** 10.1371/journal.pone.0086436

**Published:** 2014-01-17

**Authors:** Yang Liu, Dongxu Shen, Fan Zhou, Guirong Wang, Chunju An

**Affiliations:** 1 State Key Laboratory for Biology of Plant Diseases and Insect Pests, Institute of Plant Protection, Chinese Academy of Agricultural Sciences, Beijing, China; 2 Department of Entomology, College of Agriculture and Biotechnology, China Agricultural University, Beijing, China; Zhejiang University, China

## Abstract

**Background:**

The Asian corn borer (*Ostrinia furnacalis* (Guenée)) is one of the most serious corn pests in Asia. Control of this pest with entomopathogenic fungus *Beauveria bassiana* has been proposed. However, the molecular mechanisms involved in the interactions between *O. furnacalis* and *B. bassiana* are unclear, especially under the conditions that the genomic information of *O. furnacalis* is currently unavailable. So we sequenced and characterized the transcriptome of *O. furnacalis* larvae infected by *B. bassiana* with special emphasis on immunity-related genes.

**Methodology/Principal Findings:**

Illumina Hiseq2000 was used to sequence 4.64 and 4.72 Gb of the transcriptome from water-injected and *B. bassiana*-injected *O. furnacalis* larvae, respectively. *De novo* assembly generated 62,382 unigenes with mean length of 729 nt. All unigenes were searched against Nt, Nr, Swiss-Prot, COG, and KEGG databases for annotations using BLASTN or BLASTX algorithm with an E-value cut-off of 10^−5^. A total of 35,700 (57.2%) unigenes were annotated to at least one database. Pairwise comparisons resulted in 13,890 differentially expressed genes, with 5,843 up-regulated and 8,047 down-regulated. Based on sequence similarity to homologs known to participate in immune responses, we totally identified 190 potential immunity-related unigenes. They encode 45 pattern recognition proteins, 33 modulation proteins involved in the prophenoloxidase activation cascade, 46 signal transduction molecules, and 66 immune responsive effectors, respectively. The obtained transcriptome contains putative orthologs for nearly all components of the Toll, Imd, and JAK/STAT pathways. We randomly selected 24 immunity-related unigenes and investigated their expression profiles using quantitative RT-PCR assay. The results revealed variant expression patterns in response to the infection of *B. bassiana*.

**Conclusions/Significance:**

This study provides the comprehensive sequence resource and expression profiles of the immunity-related genes of *O. furnacalis*. The obtained data gives an insight into better understanding the molecular mechanisms of innate immune processes in *O. furnacalis* larvae against *B. bassiana*.

## Introduction

Innate immunity is an ancient and universal host defense mechanism found in both vertebrates and invertebrates. Insects lack adaptive immunity and mainly rely on innate immunity for defense against the invasion of pathogens or parasites [1], [2]. Innate immunity in insects consists of a humoral response based on melanization and antimicrobial peptides (AMPs), and a cellular response including phagocytosis, encapsulation, or nodulation of pathogens [2]–[6]. Melanization, involving melanin synthesis, sequesters and kills invading microorganisms or parasites [7], [8]. AMPs are directly cytotoxic to a wide range of microorganims, and are synthesized through the Toll and Imd (Immune deficiency) pathways [9]–[12].

Current understanding of insect innate immune mechanism is mainly from powerful genetic studies in *Drosophila melanogaster* [13], [14], and biochemical studies in relatively large insects, such as the silkworm, *Bombyx mori* [15], [16], the tobacco hornworm, *Manduca sexta* [17]–[19], and the beetle *Tenebrio molitor* [20], [21]. Conceptually, the innate immune reactions in insects can be divided into three steps: (1) recognition of pathogen-associated molecular patterns; (2) signal modulation and transduction; (3) production or replenishment of immune-related molecules including effectors (for review see [22], [23]). The recognition step is mediated by a group of proteins, known as pattern recognition proteins (PRPs) such as peptidoglycan recognition protein (PGRP), β-1,3-glucan recognition protein (βGRP)/gram-negative binding protein (GNBP), C-type lectin (CTL) ([24] and references within). A proteolytic cascade composed of a series of serine proteases amplifies the initial recognition signal and triggers the activation of signaling pathways [22], [25]. Ultimately, immune effectors including AMPs are induced in specific tissues, such as fat body (liver analogue) and hemocytes (insect blood cells). These effector molecules combat the invasive microorganisms in the hemolymph (insect blood), and play direct roles in insect innate immunity. So far, knowledge about the identification and involvement of immunity-related genes in various insects, especially in non-model insects, is still unclear and incomplete.

Recently, genome-wide analysis has helped to identify immunity–related genes and gene families in several insect species including 3 dipteran insects *D. melanogaster* [26], *Anopheles gambiae* [27], *Aedes agypti* [28], a hymenopteran insect *Apis mellifera* [29], a coleopteran insect *Tribolium castaneum* [30], and a lepidopteran insect *B. mori* [31]. However, the systematic analysis of immunity-related genes is hampered in other insects whose genomic information is unavailable. The introduction of novel high through-put sequencing technologies provides insight into a comprehensive immune-gene repertoire of non-model insects. Second generation high-throughput DNA sequencing platforms such as Roche 454, SOLID and Illumina combined with the development of *de novo* assembly strategies have become powerful tools in transcriptomic studies for non-model organisms without a proper reference genome, and allow targeted identification of genes which are (differentially) expressed upon activation of immune responses [32], [33]. This technology has been used, for example, to characterize the immunity-related genes in the beet armyworm *Spodoptera exigua* [34], the brown planthopper *Nilaparvata lugens* [35], and the wax moth *Galleria mellonella* [36] etc. It will greatly facilitate the future studies about analyzing the blueprint of host’s genes under microbial challenge, especially for some important insect pests in which molecular information about the immunity-related genes is lacking.

The Asian corn borer, *Ostrinia furnacalis* (Guenée), is a major insect pest in Asia and causes serious damage on corn, sorghum, millet and cotton [37]. Control of this pest with chemical insecticides is currently hindered by the cryptic nature of larval behavior. Excessive use of chemical insecticides also leads to severe environmental pollution and insecticide residence. Therefore, entomopathogenic fungi become one of promising alternates for its control. The potential for suppression of *O. furnacalis* larvae by entomopathogenic fungus *Beauveria bassiana* has been proposed [38]. However, the molecular mechanisms involved in the interactions between *O. furnacalis* and *B. bassiana* are still largely unknown, especially under the conditions that the genomic information of *O. furnacalis* is absent currently. This will greatly restrict the further development and wider adoption of entomopathogenic fungi as control agents. The first step to resolve this problem could be comprehensive identification and characterization of immunity-related genes involved in the response of *O. furnacalis* larvae against *B. bassiana*. In this study, we used the Illumina sequencing and *de novo* assembly to explore the *O. furnacalis* immune response stimulated by *B. bassiana* conidia. We obtained and characterized the transcriptome of *O. furnacalis* larvae with special emphasis on immunity-related genes. 62,382 unigenes were assembled and 35,700 were annotated to known databases. Additionally, we performed quantitative reverse transcript (qRT)-PCR analysis to compare the gene expression profiles of *B. bassiana*-infected and non-infected *O. furnacalis* larvae. All these results give us an overview of gene expression profiles of *O. furnacalis* larvae response to *B. bassiana*, and provide a shortcut for identifying new immunity genes and useful information for studying the molecular basis of host-entomopathogenic fungus interaction.

## Materials and Methods

### Insect Rearing

Asian corn borer (*O. furnacalis* (Guenée)) was kindly gifted by Dr. Kanglai He from the Institute of Plant Protection, Chinese Academy of Agricultural Sciences. The larvae were reared on an artificial diet at 28°C under a relative humidity of 70–90% and a photoperiod of 16 h light and 8 h darkness [39].

### *B. bassiana* Culture and Conidia Suspension Preparation

*B. bassiana* strain 252 was cultured on potato dextrose agar (PDA) plates at 25°C and 80% humidity. Conidia (spores) used for infection were harvested from 3–4 weeks old cultures by scraping the surface of the mycelia with sterile cell scrapers into sterile deionized water containing 0.1% Tween-80. Conidia were separated from other mycelial structures over a sterile funnel packed with autoclaved glass wool, washed twice with ddH_2_O by centrifugation at 4,000 rpm, counted and diluted to 2×10^5^ conidia/µl. Freshly prepared conidia were used for all experiments.

### Immunization and Total RNA Extraction

Three microliter of diluted conidial suspension (2×10^5^ conidia/µl) were injected into the haemocoel of *O. furnacalis* fifth instar day 0 larvae from the same batch. Injection of sterile deionized water was used as a control. After 10 h, each five larvae from challenged or control group were collected, and total RNA samples from the whole body were individually prepared using TRizol Reagent (TIANGEN, Beijing, China) following the manufacturer’s instructions. Total RNA was dissolved in H_2_O, and RNA quantity was determined on a Nanodrop ND-2000 spectrophotometer (NanoDrop products, Wilmington, DE, USA). RNA integrity was checked on Agilent 2100 BioAnalyzer (Agilent Technologies, Englewood, CO, USA).

### Library Construction and Illumina Sequencing

Ten µg of total RNA equally from 5 larvae in each group was used to isolate mRNA using oligo(dT) magnetic beads. The cDNA library of each sample was constructed using NEBNext® mRNA Library Prep Reagent Set (NEB, Ipswich, MA, USA) following the manufacturer’s protocols. Briefly, enriched poly(A) RNA of each sample was fragmented into 200–700 nt pieces with RNA Fragmentation Reagents. The cleaved RNA fragments were transcribed into the first-strand cDNA using random hexamer-primers, followed by second-strand cDNA synthesis. The resulting double-stranded cDNA (dsDNA) was purified with QiaQuick PCR extraction kit (Qiagen, Hilden, Germany) and resolved in EB buffer. The purified dsDNA was treated with T4 DNA Polymerase and T4 Polynucleotide Kinase for end-repairing and dA-tailing. After that, they were ligated to sequencing adaptors with barcode using T4 DNA ligase. Finally, fragments with around 200bp-length were purified with QiaQuick GelPurify Kit (Qiagen, Hilden, Germany), and used as templates for PCR amplification to create the cDNA library. The library was paired-end sequenced using PE90 strategy on Illumina HiSeq™ 2000 (Illumina, San Diego, CA, USA) in the Beijing Genome Institute (Shenzhen, China). The challenged and control libraries were sequenced in one lane then raw-reads were sorted out by barcodes.

### Assembly and Annotation of Transcriptomes

Raw reads from each library were filtered to remove low quality reads and the sequence reads containing adapters and poly-A/T tails. The resulting clean reads were assembled to produce unigenes using the short reads assembling program – Trinity [40]. The used parameters were as follow: min_glue  = 2, V  = 10, edge-thr  = 0.05, min_kmer_cov  = 2, path_reinforcement_distance  = 80, and group_pairs_distance  = 250. The other parameters were set as the default. Only those contigs with the length no shorter than 48 bp were used for the further assembly. The unigenes from the two samples were pooled together and clustered by TGI Clustering Tool [41]. The following parameters were used to ensure a high quality of assembly: a minimum of 95% identity, a minimum of 35 overlapping bases, a minimum of 35 scores and a maximum of 25 unmatched overhanging bases at sequence ends. The consensus cluster sequences and singletons make up the final unigene dataset.

For functional annotations, we firstly searched all unigene sequences against various protein databases such as Nr, Swiss-Prot, COG, and KEGG using BLASTX, and then searched nucleotide database Nt using BLASTN, with an E-value cut-off of 10^−5^ [42]. The BLAST results were used to extract coding region sequences (CDS) from the unigene sequences. The predicted CDS were further translated into peptide sequences. When a unigene happened to have no BLAST hits, ESTScan software [43] was used to determine the sequence direction. In addition, we performed the Gene Ontology (GO) annotations for each unigene using Blast2GO program, according to the GO associations from the BLASTX against the Nr database [44], [45].

### Identification of Differentially Expressed Genes

A mapping based on expression profile comparison was performed to examine transcript level change between control and treated sample. Clean reads of each library were mapped back to the common non-redundant dataset, respectively, using Bowtie allowing up to 3 base mismatches and a minimum length of 40 bp [46]. The expression of unigene was calculated with FPKM (Fragments per kb per million fragments) method [47]. P-values were calculated according to the hypergeometric test described by Audic and Claverie [48]. The calculated P-values were used to identify differentially expressed genes. The false discovery rate (FDR) was calculated according to the Benjamini and Hochberg algorithm [49], and further used to determine the P-value threshold in multiple testing. Genes with expression changes no less than two folds and FDRs less than 0.001 were considered as significant differentially expressed genes.

### Identification and Sequence Analysis of Immunity-related Genes from *O. furnacalis* Transcriptome

The available immunity-related gene sequences from other insect species were used as references to screen *O. furnacalis* transcriptome database obtained above. The referred insect species mainly included *A. gambiae*, *A. mellifera*, *B. mori*, *D. melanogaster*, *M. sexta*, *T. castaneum*, and *T. molitor*. The potential candidates of *O. furnacalis* immunity-related genes were confirmed by searching the BLASTX algorithm against the non-redundant NCBI nucleotide database using a cut-off E-value of 10^−5^.

For the sequence analysis of putative immunity-related genes, the deduced protein domains were determined by using Pfam (http://www.sanger.ac.uk/Software/Pfam/) and SMART (http://smart.embl.de/). Analysis of deduced amino acid sequences, including prediction of signal peptide, molecular weight and isoelectric point, was carried out in the EXPASY (Expert Protein Analysis System) proteomics server (http://www.expasy.org). Sequence comparisons and phylogenetic analysis were performed by MEGA5 software [50]. Phylogenetic trees were constructed by the neighbor-joining method, with statistical analysis by the bootstrap method, using 1000 repetitions. The phylogenetic trees were visualized in MEGA5 and colored in Adobe Illustrator (Adobe Systems).

### Quantitative Reverse Transcript (qRT)-PCR Analysis

To validate expression profile from comparative transcriptomic analysis, we designed specific primers (Table S1) to perform qRT-PCR analysis for 24 immunity-related genes. The annealing temperatures of the primers were controlled at around 62°C. Total RNA from whole body was extracted independently from 3 biological replicates as described above. DNase I-treated RNA (1 µg) was converted into first-strand cDNA using TIANScript RT Kit (TIANGEN, Beijing, China). The cDNA products were diluted 5 fold for use as template. The qRT-PCR was performed on a Applied Biosystems® 7500 Real-Time PCR System (Life Technologies, Grand Island, NY, USA) using the GoTaq® qPCR Master (Promega, Madison, WI, USA), according to the manufacturer’s instructions. *O. furnacalis* ribosomal protein L8 (*rpL8*) was used as an internal standard to normalize the expression levels. The thermal cycling conditions for qRT-PCR were 95°C for 2 min followed by 40 cycles of 95°C for 15 s, 55°C for 30 s and 68°C for 30 s ending with a melting curve generation (60°C to 95°C in increment of 0.5°C very 5 s). The relative expression of genes was calculated using 2^−ΔΔCt^ method [51].

## Results and Discussion

### Sequencing and Unigene Assembly

Parasitization of corn borer by *B. bassiana* provides a good system to study the interactions between insect hosts and entomopathogenic fungi, but the lack of genomic data of corn borer retards the relative progresses. In order to obtain detailed information about corn borer transcriptome, we subjected cDNA from *O. furnacalis* larvae with or without the infection of *B. bassiana* to Hiseq 2000 sequencing. A total of 57,411,104 and 57,669,432 raw reads were generated from water-injected (control) and *B. bassiana*-injected (treated) *O. furnacalis* libraries, respectively (Table S2). After removal of adaptor sequences, ambiguous reads, and low-quality reads (Q20<20), control and treated libraries yielded 51,594,958 (SRA accession number SRX378863) and 52,437,534 (SRA accession number SRX378865) high-quality clean reads comprised of 4,643,546,220 nucleotides (4.64 Gb) and 4,719,378,060 nucleotides (4.72 Gb), respectively (Table S2). All high-quality reads were assembled *de novo* into 95,070 (control) and 96,561 (treated) contigs with a mean length of 371 and 352 nt, respectively (Table 1). Using paired-end reads and gap-filling, these contigs were further assembled into 66,004 (mean length 588 nt) and 71,723 (mean length 511 nt) unigenes (Table 1). These two unigenes-sets were pooled for further clustering, and finally revealed a common non-redundant dataset containing 62,382 unigenes with a mean length of 729 nt, which consist of 22,889 distinct clusters and 39,493 distinct singletons (Table 1). The assembled sequences have been deposited in the NCBI Transcriptome Shotgun Assembly (TSA) Database with the title as BioProject: 228958TSA. The detailed information of each unigene, including gene ID, length, expression and functional annotation, was integrated in Table S3.

**Table 1:** Summary for the Illumina sequencing and *de novo* assembly of *O. furnacalis* transcriptome.

	Sample	Total number	Total length(nt)	Mean length(nt)	N50	Consensus sequences	Distinct clusters	Distinct singletons
**Contig**	control	95,070	35,287,785	371	706	–	–	–
	treated	96,561	34,000,293	352	580	–	–	–
**Unigene**	control	66,004	38,811,986	588	909	66,004	15,928	50,076
	treated	71,723	36,640,515	511	757	71,723	14,576	57,147
**All_unigenes**		62,382	45,459,381	729	1102	62,382	22,889	39,493

### Gene Identification, Functional Annotation and Classification

All 62,382 unigenes were annotated by searching against the databases of Nr, Nt, Swiss-Prot, KEGG, COG, and GO. As shown in Table S4, 31,277 (50.1%), 18,232 (29.2%), 22,455 (36.0%), 20,218 (32.4%), 11,462 (18.4%), and 13,451 (21.6%) unigenes were annotated in the above-referred database, respectively. The rest (26,682, 42.8%) was not annotated to the existing databases. It suggested that they were the potential sources of novel genes.

The functional annotations of all unigenes were performed mainly based on the BLASTX results against the Nr database. Among the 31,277 annotated unigenes, 15,797 (50.5%) showed strong homology (E-value smaller than 1e-45), whereas 6101 (19.5%) showed poor matches with E-value between 1e-15 and 1e-5 (Figure S1A). The similarity comparison showed 18,299 (58.5%) unigenes have more than 60% similarity with known proteins (Figure S1B). For species distribution, 19,139 (61.2%) annotated unigenes matched to *Danaus plexippus*, followed by *B. mori* (6.4%), and *T. castaneum* (3.8%) (Figure S1C). Only 246 (0.8%) unigenes matched to the known proteins in European corn borer *Ostrinia nubilalis*, an closely related specie of *O. furnacalis* (Figure S1C). One possible reason was that *O. nubilalis* genome is currently not available in NCBI comparing to the other three insect species mostly matched.

Functional classifications of all unigenes were determined by assigned to Gene Ontology (GO). GO is an international standardized gene functional classification system and covers three categories: biological process, cellular component, and molecular function. In our study, a total of 13,451 unigenes were assigned to one or more GO terms. Among these, 10,146 (75.4%) unigenes were grouped under the category of biological process, 7,510 (55.8%) under the category of cellular component, and 10,973 (81.6%) under the category of molecular function (Figure S2). The classification of GO terms was further performed at level 2 in each category. As occurred in the transcriptomes of other immune-activated insect larvae [36], the most abundant GO biological process categories were “cellular processes” (16.0%) and “metabolic processes” (12.6%). In this category, 437 unigenes comprise the subcategory of immune system process. In the cellular component category, “cell” (22.3%) and “cell part” (22.3%) represented the most abundant subcategories followed by “organelle” (14.9%). In the molecular function category, “binding” (41.4%) and “catalytic activities” (39.5%) were the most abundant (Figure S2).

We also used COG classifications to analyze the putative protein functions. In total, 11,462 unigenes were functionally classified into 25 COG categories (Figure S3). The cluster for “General function prediction only” (4,333 unigenes) constituted the largest group. The following groups were “Translation, ribosomal structure and biogenesis” (2,388 unigenes), “Replication, recombination and repair” (2,242 unigenes), and “Function unknown” (1,991, 7.3%) (Figure S3). Only 234 unigenes belonged to the group of “defense mechanisms”.

### Identification of Differentially Expressed Genes in Response to *B. bassiana* Infection

To gain insight into the global transcriptional changes taking place in *O. furnacalis* larvae infected by *B. bassiana* conidia, we performed pairwise comparisons between water-injected and *B. bassiana* conidia-injected libraries to identify the differentially expressed genes. The complete lists of differently expressed genes, including their GO and Nr annotations, are shown in Table S5. The results revealed that 13,890 unigenes exhibited significant changes after *B. bassiana* infection, including 5,843 up-regulated and 8,047 down-regulated unigenes. The detected fold changes (log2ratio) of gene expression ranged from −17.66 to 19.77.

**Figure 1:**
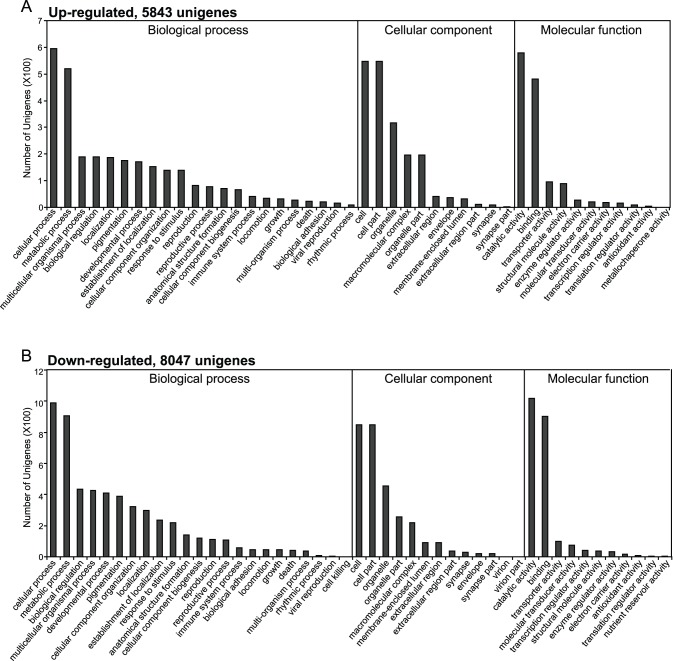
Gene ontology (GO) assignments for the enriched differently expressed unigenes after the infection of *B. bassiana*. (A) 5,843 up-regulated unigenes; (B) 8,047 down-regulated unigenes. The Y-axis shows the number of unigene in each GO term assigned at level 2.

The GO enrichment was performed to analyze the functions of all identified differently expressed genes. Among the 5,843 up-regulated unigenes, 788, 582, and 910 ones were assigned to the categories of biological process, cellular component, and molecular function, respectively. Similarly, 8,047 down-regulated unigenes were also assigned to these three categories: 1,359 in “biological process”, 943 in “cellular component”, and 1561 in “molecular function” (Figure 1 and Table S5). For both up- and down-regulated unigenes, the top 2 most abundant subcategories in each GO category were as follows: “cellular process” and “metabolic process” in “biological process” cluster, “cell” and “cell part” in the “cellular component” cluster, and “binding” and “catalytic activity” in the “molecular function” cluster (Figure 1). In the category of biological process, 42 up-regulated and 58 down-regulated genes belonged to the term of immune system process.

**Figure 2:**
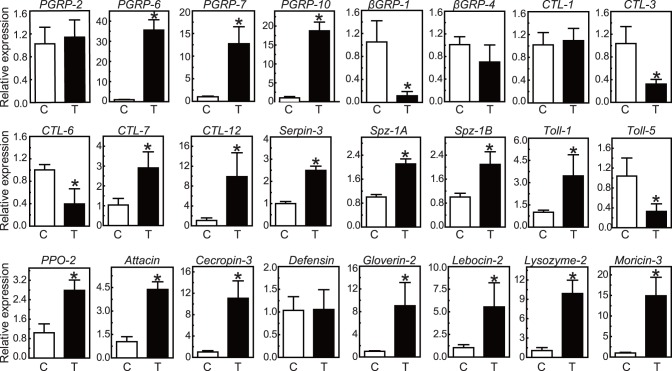
Verification of differentially expressed genes by qRT-PCR. *O. furnacalis* ribosomal protein L8 (*rpL8*) was used as an internal standard to normalize the templates. The bars represent the mean ± S.D. (*n*  = 3). Asterisks indicate means that are significantly different from the control (unpaired t test, *P*<0.05). Lack of asterisk indicates the difference is not significant (unpaired t test, *P*>0.05).

To validate the data about differently expressed gene, qRT-PCR analysis was performed using specific primers for 24 immunity-related unigenes including 12 up-regulated, 4 down-regulated and 8 unchanged unigenes. Data were presented as fold changes normalized to the *rpL8* gene in the *B. bassiana* conidia-injected sample relative to the water-injected control sample (Figure 2). Most tested unigenes exhibited the consistent expression trend in qRT-PCR analysis and the original differently expressed genes analysis (Figure 2 and Table S5). It indicates that the results of gene expression profiling from transcriptome analysis are reliable. It is worth noting that the expression profiles of 4 genes including CL1725.Contig1 (*OfCTL7*), Unigene9842 (*OfSpz-1A*), unigene13709 (*OfToll1*), and CL997.Contig1 (*OfPPO2*) were different between qRT-PCR and transcriptome analysis. In the transcriptome analysis, Unigene9842 (*OfSpz-1A*) and CL997.Contig1 (*OfPPO2*) were slightly decreased while CL1725.Contig1 (*OfCTL7*) and unigene13709 (*OfToll1*) remained unchanged (Table S5). In the qRT-PCR analysis, all four genes displayed a significant increase in transcript abundance after *B. bassiana* challenge (Figure 2). The observed differences in gene expression might be caused by the difference in the accuracy of these two assay methods. The result from the qRT-PCR analysis could be more accurate because the differently expressed genes identified in this study were from the transcriptome assembly and mapping, but not from the special DGE analysis.

### Identification of Immunity-related Genes

Insect innate immune response acts as a crucial part in defending against the infection of pathogens and parasites. In order to obtain the comprehensive perspective on the molecular biology of immune systems in *O. furnacalis*, we combined GO annotation and BLAST searches to identify the genes related with the cellular and humoral immune response. Based on molecular functions, we have divided the immune-related genes into 4 main groups: genes for signal recognition; genes involved in signal modulation and amplification; genes for signal transduction; and effector genes [52]. In total, we have identified 190 unigenes with high similarity to immunity-related genes, including 45 ones for signal recognition, 33 for signal modulation and amplification, 46 for signal transduction, and 66 for immune effectors (more details seen below). The deduced amino acid sequences of these 190 putative innate immunity genes are listed in Figure S4.

#### Genes for signal recognition

In insect innate immune response, recognition of nonself is the initial process. It is notably known that the recognition step is mediated by a group of proteins, known as pattern recognition proteins (PRPs), such as peptidoglycan recognition proteins (PGRPs), β-1,3-glucan recognition protein (βGRPs)/gram-negative binding proteins (GNBPs), C-type lectins (CTLs), scavenger receptors (SCRs) and so on [24]. In *O. furnacalis* transcriptom, we totally identified at least 45 PRP transcripts including 10 PGRPs, 4 βGRPs, 14 CTLs, 9 SCRs, 2 hemocytins, 1 hemolin, 2 galectins, 1 dscam, 1 draper and 1 eater (Table 2).

**Table 2:** Summary of the putative immunity-related unigenes identified in *O. furnacalis* transcriptome.

Designated name	Unigene ID	Nucleotide length (nt)	Full length^a^	Protein length (aa)^b^	Predicted signal peptide^c^	Fold change^d^	P value^e^	Identity to best hit (%)^f^
**Recognition**								
**PGRP**								
OfPGRP1	CL244.Contig2	2759	Yes	194	1-20G*E	3.5037	0	53
OfPGRP2	CL3377.Contig2	969	3′	286	N/A	0.2783	0.58704	65
OfPGRP3	CL6326.Contig1	1423	Yes	255		0.0976	0.73835	32
OfPGRP4	Unigene16261	1356	Yes	238	1-18G*H	7.3068	0	97
OfPGRP5	CL7407.Contig1	1638	Yes	231	1-18G*H	6.7338	0	100
OfPGRP6	Unigene20173	1042	Yes	211	1-19T*H	6.0695	0	97
OfPGRP7	Unigene287	1100	Yes	206	1-19G*F	4.4392	0	58
OfPGRP8	Unigene2928	1927	Yes	299		−1.0262	4.79E-22	53
OfPGRP9	Unigene6285	642	Yes	190	1-18S*L	1.5042	1.25E-241	98
OfPGRP10	Unigene10881	732	Yes	181	1-17T*L	5.0527	0	55
**βGRP**								
OfβGRP1	Unigene4875	1684	Yes	490	1-17A*Q	−0.5675	3.39E-115	67
OfβGRP2	CL5474.Contig2	1986	Yes	490	1-25S*V	1.2107	0	61
OfβGRP3	Unigene9808	1717	Yes	491	1-20C*R	−0.2678	9.813E-44	66
OfβGRP4	Unigene6628	1445	Yes	375	1-17A*C	1.8395	0	99
**C-type lectin**								
OfCTL1	CL106.Contig3	1408	Yes	328	1-21T*D	0.2613	1.903E-12	51
OfCTL2	Unigene5701	1211	Yes	322		0.3521	8.172E-05	52
OfCTL3	Unigene5307	1695	Yes	321	1-21S*Q	−1.3973	0	62
OfCTL4	Unigene2023	1133	Yes	321	1-17G*S	0.626	3.885E-58	39
OfCTL5	Unigene2411	1583	Yes	319		−2.5674	0	61
OfCTL6	Unigene14484	1034	Yes	314	1-22G*R	−0.2353	5.40E-87	62
OfCTL7	CL1725.Contig1	1197	Yes	307	1-20S*S	−1.3936	5.94E-26	99
OfCTL8	Unigene22572	1034	Yes	301	1-19A*Q	−0.2829	1.31E-05	47
OfCTL9	Unigene31526	1094	3′	300	N/A	−0.415	5.57E-02	50
OfCTL10	CL8286.Contig1	1072	3′	250	N/A	−2.2983	1.63E-13	94
OfCTL11	Unigene6545	1102	Yes	223	1-19A*Q	−1.5713	3.24E-204	92
OfCTL12	CL4786.Contig1	1540	Yes	220	1-19A*Q	3.8157	0	94
OfCTL13	Unigene9847	1743	Yes	207	1-21A*Q	−3.5946	0	87
OfCTL14	CL321.Contig3	763	Yes	184	1-21T*V	−0.1434	0.68032	88
**galectin**								
OfGalectin1	CL1314.Contig2	1433	Yes	365		0.1771	3.65E-01	67
OfGalectin2	Unigene31759	1011	Yes	255		1.9018	5.87E-96	41
**scavenger receptor (SCR)**								
OfSCR1	CL3884.Contig1	2733	Yes	737		0.3777	0.039813	56
OfSCR2	Unigene3524	2348	Yes	626		−0.6523	2.98E-09	75
OfSCR3	Unigene4658	2326	Yes	566		0.1914	1.47E-02	60
OfSCR4	Unigene12877	1689	5′	521		−0.2322	5.40E-05	33
OfSCR5	CL2453.Contig1	2411	Yes	515		−0.031	7.61E-01	89
OfSCR6	Unigene18387	1692	5′	511		−2.9157	8.78E-36	41
OfSCR7	CL675.Contig2	2033	Yes	496		0.3464	2.34E-07	67
OfSCR8	Unigene3062	1832	Yes	439	1-16A*Q	−2.9802	0	53
OfSCR9	Unigene3589	1450	3′	425	N/A	−4.8973	3.78E-69	36
**hemocytin**								
OfHemocytin1	Unigene72	4646	3′	1497	N/A	−3.2442	0	61
OfHemocytin2	Unigene2798	2134	M	710	N/A	−2.8968	2.45E-41	43
**hemolin**								
OfHemolin	Unigene9924	1649	Yes	456		2.7711	0	47
**dscam**								
OfDscam	CL3756.Contig3	3280	M	1093	N/A	−1.4098	5.39E-44	85
**Draper**								
OfDraper	Unigene5316	3199	5′	954	1-17A*L	−0.5145	6.75E-21	53
**Eater**								
OfEater	Unigene6859	2266	Yes	664	1-19S*D	−1.5664	2.19E-35	41
**Modulation**								
**Clip-domain serine protease**								
OfSP1	CL399.Contig2	1992	Yes	358	1-20A*Q	−0.7183	2.12E-08	56
OfSP2	Unigene18713	1454	Yes	421	1-18G*A	0.2364	5.82E-19	57
OfSP3	CL5234.Contig1	1602	Yes	400	1-21A*Q	−0.8512	1.3E-10	54
OfSP4	Unigene13481	1579	Yes	444	1-17S*S	0.2868	5.13E-06	67
OfSP5	CL1110.Contig5	1381	Yes	385	1-17A*E	−0.6014	3.69E-03	34
OfSP7	CL2452.Contig1	1865	Yes	421	1-19G*Q	1.7753	0	44
OfSP8	CL4641.Contig3	2105	Yes	424	1-19G*Q	1.4516	0	63
OfSP10	Unigene941	1669	Yes	400	1-18A*N	−1.0384	4.13E-297	99
OfSP12	Unigene3077	1873	Yes	370	1-21A*Q	1.0886	0	64
OfSP13	Unigene17162	1338	Yes	380	1-19T*Q	1.2121	1.56E-296	67
OfSP14	Unigene21915	1830	Yes	451		0.1695	1.01E-08	57
OfSP17	Unigene21394	2158	Yes	530	1-26S*E	−2.6323	0	55
OfSP37	CL5321.Contig1	1380	Yes	389	1-16G*D	−2.3145	1.40E-101	70
**Clip-domain serine protease homolog (SPH)**								
OfSPH8	Unigene10046	1469	Yes	411	1-15A*Q	0.6276	2.22E-196	62
OfSPH9	CL945.Contig1	2031	Yes	604	1-17A*D	−2.2505	1.77E-203	43
OfSPH10	Unigene12563	1321	Yes	399	1-20G*Q	−1.2335	0	36
**serpin**								
OfSerpin-1A	CL2978.Contig1	1497	Yes	397	1-16A*D	−0.0006	9.95E-01	58
OfSerpin-1B	CL2978.Contig2	1564	Yes	406	1-16A*D	−0.097	1.35E-01	59
OfSerpin-1C	CL2978.Contig3	1450	Yes	393	1-16A*D	−1.3272	2.89E-235	58
OfSerpin-1D	Unigene18304	2181	Yes	395	1-16A*D	−0.0419	2.92E-01	62
OfSerpin-2	CL2637.Contig5	1544	Yes	377		0.9159	5.328E-16	69
OfSerpin-3	CL7904.Contig2	1770	3′	375	N/A	2.3578	0	68
OfSerpin-4	CL9195.Contig5	1561	Yes	413	1-18G*Q	4.7853	0	63
OfSerpin-5	CL5373.Contig1	1478	Yes	395	1-16C*A	−1.2879	3.16E-219	72
OfSerpin-6	CL5354.Contig1	1570	3′	295	N/A	1.6927	1.24E-163	88
OfSerpin-7	CL3593.Contig1	2276	Yes	719	1-21A*G	−2.9453	0	55
OfSerpin-8	CL60.Contig2	2034	Yes	646	1-20A*D	−2.6132	4.20E-60	39
OfSerpin-9	CL3162.Contig2	2540	Yes	542	1-18A*Q	0.0382	4.08E-02	55
OfSerpin-10	Unigene7142	1880	Yes	518	1-17A*R	−2.0645	3.48E-118	73
OfSerpin-11	CL2102.Contig3	2110	Yes	457	1-19P*R	−0.3346	5.16E-06	63
OfSerpin-12	Unigene868	1592	Yes	428	1-23G*G	1.0433	3.26E-124	68
OfSerpin-13	Unigene17483	1377	Yes	406	1-20G*Q	−0.5347	6.74E-37	51
OfSerpin-14	Unigene9805	1011	3′	296	N/A	0.8088	1.88E-06	48
**Transduction**								
**(Toll pathway)**								
**Spätzle**								
OfSpz1A	Unigene9842	1126	Yes	319	1-17A*Y	−0.367	3.26E-03	33
OfSpz1B	Unigene6173	773	Yes	221	1-18A*Y	−0.0271	8.16E-01	32
OfSpz3	Unigene6845	1539	3′	481	N/A	−0.308	1.58E-01	73
OfSpz4	Unigene18729	1291	5′	413	1-17A*Y	2.1258	5.77E-20	62
OfSpz5	Unigene27639	1584	Yes	418	1-28G*Y	−3.3208	3.13E-26	33
OfSpz6	Unigene7136	1322	3′	398	N/A	1.9615	3.19E-11	56
**Toll**								
OfToll1	Unigene13709	4736	Yes	1340	1-22G*A	−0.227	5.67E-04	89
OfToll2	CL3792.Contig1	3426	Yes	1003	1-22A*C	−1.1004	7.55E-89	32
OfToll3	Unigene7123	2672	Yes	770	1-22C*S	−0.4329	8.24E-06	36
OfToll4	CL1140.Contig1	2841	Yes	745	1-19G*F	−0.0957	1.70E-01	51
OfToll5	Unigene9148	2622	3′	737	N/A	−2.2303	6.64E-57	79
OfToll6	CL8435.Contig1	2420	3′	709	N/A	−0.5819	3.94E-04	53
OfToll7	Unigene150	1832	3′	588	N/A	−1.69	3.61E-13	95
OfToll8	Unigene16267	1800	Yes	425	1-19G*E	−0.993	4.92E-26	29
OfToll9	Unigene16850	1350	3′	417	N/A	−1.0493	7.53E-84	40
OfToll10	Unigene5256	1227	M	409	N/A	−0.3618	1.05E-01	87
OfToll11	Unigene2699	1022	3′	320	N/A	0.0403	8.61E-01	84
OfToll12	Unigene13736	917	M	305	N/A	−1.9247	1.19E-07	80
**MyD88**								
OfMyD88-1	Unigene142	2405	Yes	379		−0.2001	4.00E-02	42
OfMyD88-2	CL1575.Contig1	1567	Yes	378		0.5748	1.73E-13	59
**Tube**								
OfTube	Unigene3838	427	5;	94		−0.6087	1.15E-02	39
**Pelle**								
OfPelle1	Unigene9421	2053	3′	507	N/A	−0.6058	1.14E-09	29
OfPelle2	CL1687.Contig1	2390	Yes	483		0.6875	1.27E-16	40
**Pellino**								
OfPellino	CL6004.Contig1	2131	Yes	432		−0.0473	5.99E-01	74
**TRAF2**								
OfTRAF2	Unigene19082	1146	5′	358		−0.7026	2.76E-03	79
**Cactus**								
OfCactus	CL1201.Contig1	3009	Yes	335		3.0653	0	48
**Dorsal/Dif**								
OfDorsal	CL7451.Contig1	2888	Yes	615		0.2955	2.26E-03	50
**Tollip**								
OfTollip	CL951.Contig1	1116	Yes	276		−0.3531	1.23E-02	62
**(Imd pathway)**								
**Imd**								
OfImd	CL2356.Contig1	1122	Yes	253		0.4303	9.28E-07	48
**Dredd**								
OfDredd	CL978.Contig1	1474	5′	427		0.1654	2.92E-02	42
**FADD**								
OfFADD	Unigene6623	860	Yes	221		−0.0392	5.36E-01	45
**relish**								
OfRelish	CL6686.Contig2	3768	Yes	956		1.916	0	52
**caspar**								
OfCaspar	CL1175.Contig1	2264	3′	665	N/A	−0.5822	5.67E-06	41
**IKK**								
OfIKKγ	Unigene2344	3484	Yes	681		2.742	0	20
OfIKKβ	CL495.Contig1	1924	5′	613		0.1559	2.82E-01	34
**Tak1**								
OfTak1	Unigene9186	875	M	291	N/A	−1.0067	1.78E-04	72
**IAP2**								
OfIAP2	CL9344.Contig1	1872	Yes	566		1.0766	2.09E-07	56
**Tab2**								
OfTab2	CL6310.Contig2	2353	Yes	592		0.5095	4.04E-03	65
**(JAK/STAT pathway)**								
**STAT**								
OfSTAT	CL7684.Contig1	2964	3′	721	N/A	−0.419	1.44E-04	83
**Domeless**								
OfDomeless	Unigene9848	3041	5′	919		0.9573	4.25E-30	29
**PIAS**								
OfPIAS1	CL862.Contig4	2465	Yes	653		0.0151	9.76E-01	76
OfPIAS2	CL862.Contig5	2983	Yes	619		−0.6311	9.07E-03	77
OfPIAS3	CL862.Contig6	3033	Yes	614		0.2136	7.71E-01	76
OfPIAS4	CL862.Contig3	2181	Yes	580		−1.2089	3.99E-03	77
**SOCS**								
OfSOCS	Unigene9103	1543	Yes	354		−0.5117	3.38E-03	81
**Hopscotch**								
OfHopscotch	Unigene16260	1097	5′	323		−1.0724	4.60E-04	38
**Effectors**								
**Prophenoloxidase (PPO)**								
OfPPO1	CL5552.Contig1	1299	5′	407		−1.9558	2.20E-74	77
OfPPO2	CL997.Contig1	2366	Yes	692		−2.296	6.45E-207	100
OfPPO3	Unigene28348	1153	5′	365		−1.3114	5.38E-28	70
**(Antimicrobial peptide, AMP)**								
**lysozyme**								
OfLys1	Unigene9475	2426	Yes	155	1-25G*Q	−0.5278	5.215E-12	72
OfLys2	CL5427.Contig1	905	Yes	140	1-20A*K	2.6495	0	99
OfLys3	Unigene20582	1732	Yes	140	1-20A*V	2.1514	0	87
OfLys4	CL6947.Contig5	523	5′	137	1-16G*V	−3.0725	1.59E-02	69
OfLys5	CL5801.Contig1	1027	Yes	143	1-20G*R	0.949	1.32E-16	51
OfLys6	Unigene13076	827	Yes	140	1-18A*F	−2.4845	0	66
OfLys-L1	Unigene10659	780	Yes	186	1-17G*R	0.2146	1.07E-01	68
OfLys-L2	CL9850.Contig2	1877	Yes	180	1-22S*T	−0.4783	4.10E-14	72
**cecropin**								
OfCec1	Unigene13269	547	Yes	68	1-22A*T	2.7295	0	51
OfCec2	CL1178.Contig1	2056	Yes	66	1-22A*A	2.7421	0	73
OfCec3	CL7041.Contig1	723	Yes	66	1-22A*A	2.1301	0	66
OfCec4	Unigene828	1332	Yes	65	1-22A*S	3.705	0	58
OfCec5	Unigene14482	1186	Yes	64	1-22A*A	3.0652	0	75
OfCec6	Unigene22346	470	Yes	61	1-22A*A	2.8013	0	78
**defensin**								
OfDefensin	CL4424.Contig1	477	Yes	108	1-22S*F	0.6963	9.80E-61	33
**attacin**								
OfAttacin	CL8975.Contig1	983	Yes	204	1-17S*Q	1.4461	0	52
**gloverin**								
OfGloverin1	Unigene1854	1164	Yes	196	1-15A*Q	1.5723	0	57
OfGloverin2	CL516.Contig4	1384	Yes	198	1-15A*Q	1.2401	0	57
**Lebocin**								
OfLebocin1	CL8338.Contig1	1310	Yes	199	1-22A*Q	2.4102	0	51
OfLebocin2	CL8528.Contig2	391	5′	124	1-22A*Q	1.7827	0	37
OfLebocin3	CL8528.Contig1	386	5′	121	1-22A*Q	1.285	0	37
**Moricin**								
OfMoricin1	Unigene8386	388	Yes	63	1-23A*N	4.0096	0	58
OfMoricin2	Unigene4140	359	Yes	63	1-22A*N	3.0459	0	53
OfMoricin3	CL4055.Contig5	536	Yes	62	1-23A*N	4.3116	9.00E-304	66
OfMoricin4	Unigene33399	239	5′	61	1-23G*N	3.4761	6.63E-12	50
**Nitric oxide synthase (NOS)**								
OfNOS1	CL332.Contig1	2618	3′	777	N/A	1.1857	3.31E-16	82
OfNOS2	Unigene9115	1901	3′	575	N/A	−0.1862	3.61E-01	90
**reeler**								
OfReeler	CL9361.Contig1	1064	Yes	165	1-18G*Y	4.8393	0	42
**NADPH oxidase (NOX)**								
OfNOX	Unigene18492	1617	M	538	N/A	−0.6201	0.0001625	59
**Peroxidase (Pox)**								
OfPox1	CL6829.Contig2	4356	Yes	1341	1-24A*Q	−0.3948	2.338E-06	60
OfPox2	Unigene13729	3539	Yes	798		−0.1627	1.416E-06	74
OfPox3	CL812.Contig1	2051	Yes	648	1-17G*N	−2.1365	4.55E-232	54
OfPox4	Unigene2378	2149	Yes	656	1-17G*G	2.4212	1.85E-123	55
OfPox5	CL517.Contig7	2522	Yes	716	1-16A*L	−1.9023	7.623E-08	53
OfPox6	Unigene5009	1124	M	374	N/A	−2.5073	3.684E-11	53
OfPox7	Unigene26669	1049	3′	331	N/A	−11.7799	9.637E-11	69
OfPox8	Unigene6370	933	3′	275	N/A	−2.7268	2.448E-51	48
OfPox9	Unigene19867	818	M	272	N/A	0.4346	0.165	46
OfPox10	Unigene21448	821	3′	257	N/A	0.4738	0.089999	50
OfPox11	Unigene935	1302	Yes	249	1-19C*T	−0.5707	4E-23	92
OfPox12	CL1677.Contig1	1085	Yes	227		1.6154	1.459E-06	82
OfPox13	Unigene4211	991	Yes	223		−0.082	0.37496	83
OfPox14	CL9355.Contig4	1490	Yes	198	1-18T*A	−0.7768	0.0084466	79
OfPox15	CL9299.Contig1	1357	Yes	195		0.3196	4.936E-11	95
OfPox16	CL3991.Contig2	684	3′	166	N/A	0.501	0.14413	43
OfPox17	Unigene5118	571	M	190	N/A	−0.9783	0.0004929	46
**Superoxide dismutase (SOD)**								
OfSOD1	Unigene16806	1130	Yes	286		0.4061	0.0059053	65
OfSOD2	Unigene22638	1010	Yes	241	1-18A*L	0.5179	0.024544	77
OfSOD3	Unigene4403	915	Yes	216		−0.6654	9.254E-54	85
OfSOD4	Unigene18267	621	5′	192	1-19A*D	−2.4673	3.312E-95	55
OfSOD5	CL5843.Contig2	1218	Yes	172	1-16A*H	−2.2343	0	64
OfSOD6	CL9771.Contig2	1349	Yes	154		−0.0839	0.058992	89
**Catalase**								
OfCAT	CL2411.Contig1	2051	Yes	550		−0.6635	1.57E-203	93
**Thioredoxin reductase**								
OfTrxR	CL3171.Contig1	1872	Yes	494		−0.1048	0.41271	71
**Thioredoxin**								
OfTrx1	Unigene21754	1356	Yes	286		−0.535	5.03E-35	86
OfTrx2	CL5990.Contig3	953	Yes	153		−0.0055	0.98746	85
OfTrx3	CL6996.Contig1	1655	Yes	150		−0.5278	3.946E-11	84
OfTrx4	CL9041.Contig1	1238	Yes	106		0.4083	2.998E-38	97
**Peroxiredoxin**								
OfPrx1	Unigene935	1302	Yes	249	1-19C*T	−0.5707	4E-23	92
OfPrx2	CL1677.Contig1	1085	Yes	227		1.6154	1.459E-06	80
OfPrx3	Unigene4211	991	Yes	223		−0.082	0.37496	84
OfPrx4	CL9299.Contig1	1357	Yes	195		0.3196	4.936E-11	92
OfPrx5	CL8502.Contig1	1777	Yes	185		−1.4136	1.854E-73	83

^a^ The mark of Yes, 5′, 3′, and M means that the fragment of the unigene consists of complete open reading frame, 5′-end containing start codon, 3′-end containing stop codon, and the middle part without start and stop codons, respectively.

^b^ The number indicates the length of amino acid sequences deduced from available coding region, which is complete or not.

^c^ Blank, signal peptide unpredictable. N/A, truncated at amino-terminus.

^d^ The fold change is calculated by log2 (treated_RPKM/control_RPKM). The value no less than 1 or no more than −1 is defined as up-regulated or down-regulated, respectively.

^e^ P-value is determined by FDR (false discovery rate).

^f^ Identity indicates the percentage of identical amino acid residues in *O. furnacalis* unigenes to the sequence best hit in BLASTX.

**Figure 3:**
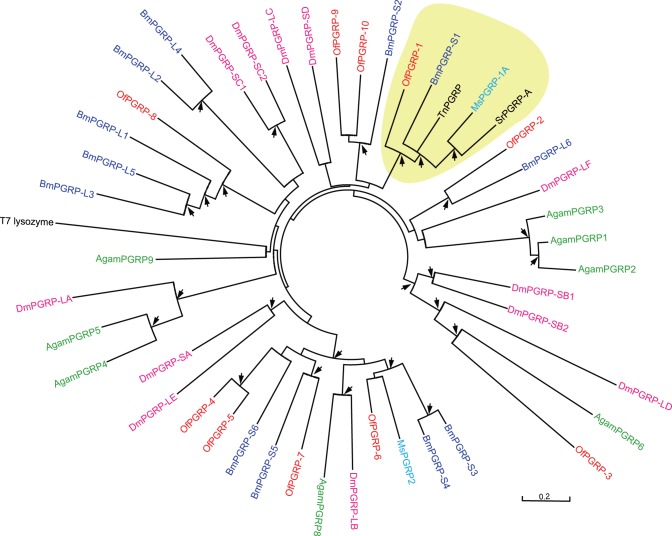
Phylogenetic analysis of peptidoglycan recognition proteins (PGRPs). The amino acid sequences from 10 *Ostrinia* (Of, red), 12 *Drosophila* (Dm, pink), 8 *Anopheles* (Ag, green), 12 *Bombyx* (Bm, purple), 2 *Manduca* (Ms, blue), one *Samia ricini* (Sr, black), one *Trichoplusia ni* (Tn, black) PGRPs, and T7 lysozyme (black) were used to build the unrooted tree. The clade that groups OfPGRP1 with other PGRPs known as recognition receptors in the PPO activation cascade (BmPGRP-S1 and MsPGRP-1A) was shaded in yellow. The arrows at nodes denote bootstrap value greater than 700 from 1000 trials.

PGRPs play a central role for the recognition of invading microorganisms in insect immunity by specifically binding to and hydrolyzing bacterial peptidoglycan [53], [54]. The first PGRP was isolated from hemolymph of the silkworm, as a pattern recognition receptor to trigger prophenoloxidase (PPO) activation cascade [53]. All PGRP family members share at least one conserved PGRP domain, with similarity to bacteriophage T7 lysozyme, a zinc-dependent N-acetylmuramoyl-L-alanine amidase [55]. The most highly diversified PGRP homologues have been identified in *Drosophila*. *Drosophila* has 13 PGRP genes encoding 19 proteins, which are classified into short (S) and long (L) forms [55], [56]. Among 19 *Drosophila* PGRPs, six (DmPGRP-SB1/−SB2/−SC1a/−SC1b/−SC2/−LB) have amidase activity, and five (DmPGRP-SA/−SD/−LC/−LE/−LF) lack amidase activity but function as receptors to activate immune signaling pathways [55]. In this study, we identified 10 putative PGRP sequences and designated them as *OfPGRP1-10*. With the exception of *OfPGRP2*, the other 9 PGRP transcripts were predicted to be full-lengthed (Table 2). Alignment of 10 putative *O. furnacalis* PGRPs with *Drosophila* PGRPs and T7 lysozyme indicated that the deduced amino acid sequences of OfPGRP1-3 and OfPGRP8-10 lack at least one of five active site residues essential for amidase activity in T7 lysozyme (H17, Y46, H122, K128 and C130, K128 is replaced by T in *Drosophila* PGRPs). It suggests that these six *O. furnacalis* PGRPs potentially act as receptors for peptidoglycan to initiate a signaling pathway while the left 4 ones (OfPGRP4-7) theoretically have amidase activity and might serve as an intracellular peptidoglycan scavenger. Bootstrap analysis reveals that OfPGRP1 is an ortholog of *M. sexta* PGRP-1 and *B. mori* PGRP-S1 which both have been verified to function as recognition receptors in the PPO activation cascade [53], [54] (Figure 3). It suggests that OfPGRP1 may also act as a peptidoglycan receptor in activating PPO cascade upon the challenge of *B. bassiana*. Moreover, the analysis of digital expression profile showed that 7 out of 10 identified PGRPs (*OfPGRP1*, *4–7*, 9 and *10*) were obviously up-regulated after the infection of *B. bassiana*, whereas the other 3 *OfPGRPs* remained unchanged (Table 2 and Table S5). We randomly selected 4 PGRP genes (*OfPGRP2*, *6*, *7* and *10*) to analyze their transcript changes after the injection of *B. bassiana* using qRT-PCR methods. *OfPGRP-6*, *-7* and *-10* showed very high expression levels in *B. bassiana*-injected corn borer larvae, while *OfPGRP2* mRNA level was consistent (Figure 2). We speculated that *O. furnacalis* PGRPs might play different roles, and work in concert with each other to defend against the invasion of *B. bassiana*.

**Figure 4:**
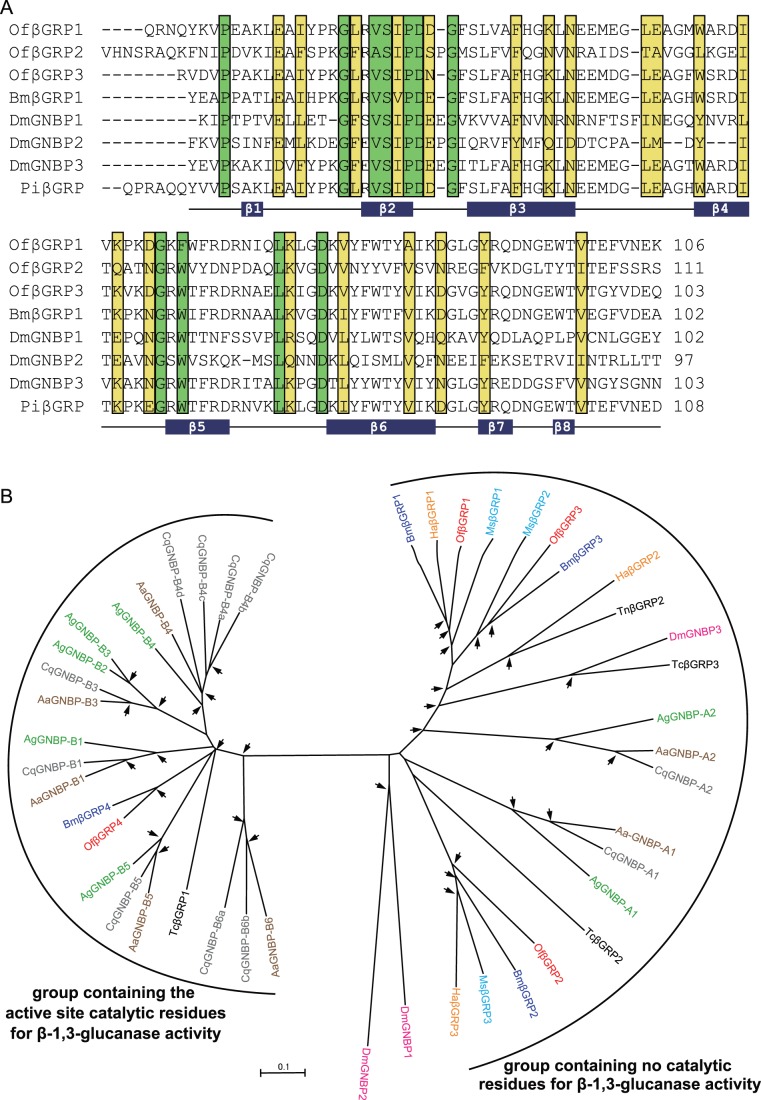
Alignments of the N-terminal domains of β-glucan recognition proteins (βGRPs). **(A)** The deduced amino acid sequences of *O. furnacalis* βGRP1-3 (OfβGRP1-3) were compared with *Drosophila* GNBP1-3 (DmGNBP1-3), *Bombyx* βGRP1 (BmβGRP1) and *Plodia interpunctella* βGRP (PiβGRP). The amino acids in green and yellow shade indicate the conserved and type-conserved residues, respectively. The predicted secondary structural elements of eight β-strands are shown below the alignments. **(B) Phylogenetic relationships of βGRPs**. The aligned sequences of βGRP family members were from *Ostrinia* (Of, red), *Drosophila* (Dm, pink), *Anopheles* (Ag, green), *Aedes* (Aa, brown), *Culex quinquefasciatus* (Cq, grey), *Bombyx* (Bm, purple), *Manduca* (Ms, blue), *Helicoverpa armigera* (Ha, orange), *Trichoplusia* (Tn, black), and *Tribolium* (Tc, black). Two divided groups are indicated by brackets. For explanation of the arrows see Fig. 6.

βGRP/GNBP belongs to another pattern recognition protein family. This family contains two functionally different proteins, one of which has a strong affinity to β-1,3-glucans of fungal cell walls (βGRP) and the other is dubbed as Gram-negative binding protein (GNBP) but binds to Gram-negative bacteria or Gram-positive bacteria [57]. Since the first βGRP was identified in the PPO-activation system of *B. mori* [58], βGRPs have been identified in insects including *Drosophila* (3 genes) [57], *Anopheles* (7 genes) [27], *Apis* (2 genes) [29], *Manduca* (2 genes) [59], [60], and *Tribolium* (3 genes) [30]. They all consist of a conserved N-terminal β-1,3-glucan-recognition domain for the detection of pathogens or parasites, and a C-terminal glucanase-like domain with undefined function [61], [62]. Three-dimensional structures of BmβGRP1 and DmGNBP3 further revealed that the N-terminal β-1,3-glucan-recognition domain actually adopts a β-sandwich structure formed by eight β-strands [62], [63]. In this study, we identified 4 *βGRP/GNBP* genes with predicted full length, and designated them as *OfβGRP1-4*. All OfβGRPs are assumed to be secreted proteins because they have putative signal peptides (Table 2). A comparison of the deduced amino acid sequences with *Drosophila* GNBP1-3 and *Bombyx* βGRP1 showed that OfβGRP1-3 contained the putative N-terminal β-1,3-glucan-recognition domain and the C-terminal glucanase-like domain, but OfβGRP4 lacked the N-terminal β-1,3-glucan-recognition domain, suggesting that it is possibly unable to directly bind to β-1,3-glucan. Additionally, high sequences similarities were observed in the N-terminal domains of deduced *O. furnacalis*, *Drosophila* and *Bombyx* homologues. OfβGRP1-3 also comprise eight conserved β-strands (Figure 4A), suggesting their ability to bind to β-1,3-glucan. We performed the phylogenetic analysis for all OfβGRP1-4 and 42 βGRP sequences from other insect species to investigate the relationship between *O. furnacalis* βGRPs and others. As shown in Figure 4B, all 46 βGRPs were clustered into two groups, one containing the active catalytic residues for β-1,3-glucanase activity and the other containing no such residues. OfβGRP1-4 is presented as 1∶1 orthologs to *B. mori* βGRP1-4. The orthologs for OfβGRP1, OfβGRP2 and OfβGRP3 are present in lepidopterans but not in dipterans (Figure 4B). *OfβGRP1* and *OfβGRP3* are predicted to be paralogs and have expanded from a common ancestral gene after Lepidoptera had diverged (Figure 4B). The digital expression profiles showed *OfβGRP2* and *OfβGRP4* mRNA level slightly increased while *OfβGRP1* and *OfβGRP3* were consistent in response to the infection of *B. bassiana* (Table 2). The qRT-PCR assay showed *OfβGRP1* mRNA level decreased and *OfβGRP4* expression kept unchanged after challenge (Figure 2).

**Figure 5:**
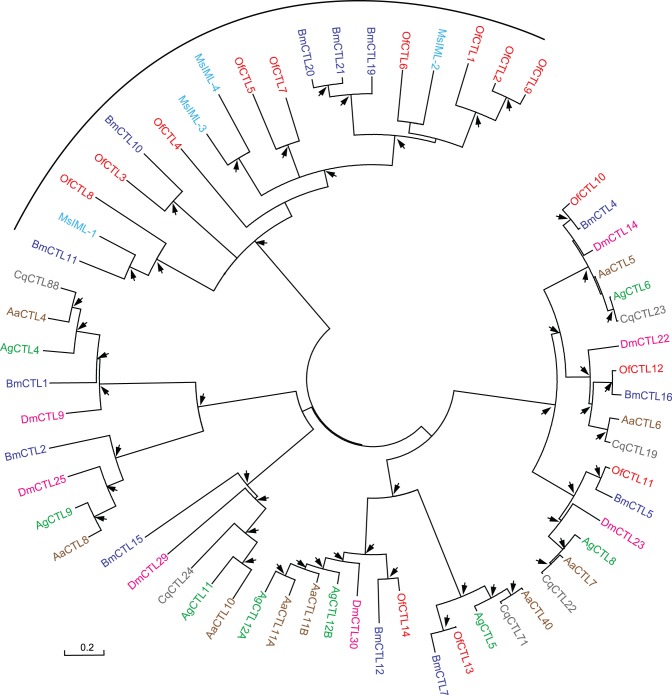
Phylogenetic analysis of C-type lectins (CTLs). The amino acid sequences of 61 CTLs from *Ostrinia* (Of, red), *Drosophila* (Dm, pink), *Anopheles* (Ag, green), *Aedes* (Aa, brown), *Culex* (Cq, grey), *Bombyx* (Bm, purple), and *Manduca* (Ms, blue) were examined. Lepidopteran-specific CTLs are indicted with bracket. For explanation of the arrows see Fig. 6.

C-type lectin (CTL) is probably the largest lectin family. They are a group of soluble and membrane-bound proteins that associate with carbohydrates in a Ca^2+^-dependent manner [64]. Invertebrate CTLs participate in immune responses including PPO activation [65], hemocyte-mediated nodule formation and encapsulation [66], opsonization and microbial clearance [67]. CTLs have a characteristic carbohydrate-binding domain (CRD) with a well-defined structure stabilized by two or three pairs of disulfide bonds [65]. Insect CTLs normally consist of tandem CRDs. In this study, we identified 14 CTL genes, and designated them as *OfCTL1-14* (Table 2). All deduced *O. furnacalis* CTLs contain two consecutive CRDs, a characteristic for lepidopteran CTLs (Figure 5). Although N- and C-terminal CRDs from *Hyphantria cunea* lectin are reported to have different sugar-binding specificities, the detailed function of these CRDs is not yet clarified [68]. We postulated that the two CRDs of *O. furnacalis* CTLs also have different sugar-binding specificity and bind to different microorganisms. Except for CRDs, other structural modules have been identified in some CTLs. For example, *Drosophila* Furrowed (a *Drosopohila* CTL) and *Bombyx* CTL2 have Sushi motifs and transmembrane domain in addition to CRD [31], [69]. We only found CRDs in OfCTL1-14 sequences. There might be some unknown CTLs we did not identify in the transcriptome. OfCTL6 has 56% amino acid sequence identity to *M. sexta* IML-2 which binds to lipopolysaccharide to stimulate hemocyte encapsulation and melanization [64], [65]. This suggests that OfCTL6 might also play an important role as recognition receptor during the early phase of microbial infection. Phylogenetic analyses indicated that OfCTL1-9 only clustered with lepidopteran CTLs, and OfCTL10-14 grouped with both lepidopteran and dipteran CTLs (Figure 5). qRT-PCR assay revealed that the transcript level of *OfCTL7* and *OfCTL12* increased, *OfCTL3* and *OfCTL6* decreased, whereas *OfCTL1* remained unchanged (Figure 2).

In addition, we also identified other PRP genes from *O. furnacalis* transcriptome, including 9 *scavenger receptors* (SCRs), 2 *hemocytins*, 1 *hemolin*, 2 *galectins*, 1 *dscam*, 1 *Draper* and 1 *Eater* (Table 2). The identification of a large number of PRP genes laid a good foundation for the further cloning and functional studies of PRPs in *O. furnacalis*.

#### Genes for extracellular signal modulation and amplification

After infectious parasites or pathogens are recognized by insects, the invasive signals are modulated by extracellular cascades. Similar to the coagulation pathway and complement system in human, insect plasma factors such as serine proteases (SPs) and serine protease homologs (SPHs) play critical roles in signal relaying/tuning and execution mechanisms [31], [70]. SPs typically contains a catalytic triad consisting of His, Asp, and Ser residues, which are embedded in highly conserved sequence motif of TAAHC, DIAL and GDSGG, respectively [71]. SPHs lack proteolytic activity due to the substitution of the catalytic triad residues, but they can enhance specificities and catalytic activities of SPs [72]-[74]. SPs and SPHs constitute one of the largest protein families in insects [27], [75], [76]. We have identified 121 potential SP and SPH transcripts in *O. furnacalis* transcriptome. Given that the catalytic triad is vital to define a SP or SPH, we manually checked the deduced amino acid sequences of all 121 transcripts, and only kept those containing all three sequence motifs. Finally we obtained 47 SP (designated as OfSP1-OfSP47) and 14 SPH (designated as OfSPH1-OfSPH14) transcripts. Fifty-six genes are predicted to have full length, 18 of which encode polypeptides with a SP or SP-like domain and other structural modules. These include 13 SPs (SP1-SP5, SP7, SP8, SP10, SP12-SP14, SP17 and SP37) and 3 SPHs (SPH8-SPH10) which contain one or more regulatory clip domains (Table 2), one SP (SP40) which contains a CUB domain, one SP (SP46) which contains five low density lipoprotein receptor A repeats (LDLa) domains and two complement control protein (CCP) domains. The clip domain is an important structural unit in which six conserved cysteine residues form three disulfide bonds [25], [77]. In arthropods, clip-domain SPs, and occasionally clip-domain SPHs, are involved in many immune signaling pathways, such as melanization cascade and Toll pathway [31], [71], [76]. In 13 SPs with clip domain, SP1 and SP13 mediated the immune responses of corn borer against *B. bassiana* by participating in the PPO activation cascade (submitted to Amino Acids). The other 11 clip-SPs are still under the investigation. Moreover, it is noteworthy that *O. furnacalis* SP46, with a large size and complex domain structure, is most similar to *M. sexta* HP14 [78] and *T. molitor* MSP [21] which both function as an initial enzyme to be recruited into the recognition complex in PPO activation cascade. We, therefore, inferred that SP46 also acts as the first enzyme in a serine protease pathway. Phylogenetic analysis indicated that *O. furnacalis* clip-domain proteins are divided into four subfamilies (Figure S5). The subfamily A is composed of SPHs solely while subfamilies B, C and D comprise SPs mainly. The four groups of SP-related genes may represent lineages derived from ancient evolutionary events since similar subfamilies also existed in *Anopheles* [27], *Drosophila* [75], and *Tribolium* [30].

Extracellular serine protease cascade are often regulated by members of the serine protease inhibitor (serpin) superfamily [79], [80]. Serpins contain ∼400 amino acid residues with an exposed reactive-center loop near their carboxyl terminus [81]. They inhibit serine proteases by forming covalent complexes, and therefore function as suicide-substrate inhibitors. Serpins have been reported to participate in the regulation of melanization reactions in three different insect orders (flies, beetle, and moths), including *Anopheles* SRPN2 [82], [83], *Drosophila* Spn27A [84], [85], *Manduca* serpin-3 [86], [87], and *Tenebrio* SPN40, SPN55 and SPN48 [88]. In this study, we have identified 17 serpin transcripts from *O. furnacalis* transcriptome, which were designated as *OfSerpin1A-1D, and 2 through 14* (Table 2). This includes four splicing variants for *OfSerpin-1*, in which only the last 40-53 amino acid residues in the reactive center loop are variable. Fourteen out of 17 serpin transcripts contain complete open reading frame with the exception of *serpin-3*, *serpin-6*, and *serpin-14*. Among 14 complete serpins, 13 ones consisted of a predicted signal peptide, suggesting they are secreted proteins. Serpin-2 lacks a secretion signal sequence, suggesting it might be an intracellular protein. In the phylogenetic analysis, OfSerpin1-6s are presented as 1∶1∶1 orthologs to *Manduca* serpin1-6 and *Bombyx* serpin1-6, suggesting that *O. furnacalis* serpin1-6 has immune functions similar to *Manduca* and *Bombyx* serpins (Figure S6). It is noteworthy that one clade includes *O. furnacalis* serpin-3 as well as *Anopheles* SRPN2, *Aedes* Serpin-2, *Drosophila* Spn27A, *Manduca* serpin-3, and *Bombyx* serpin-3, which all functions as inhibitors of the melanization cascade [82], [84]-[86]. Thus OfSerpin-3 may also regulate serine protease(s) involved in the melanization reaction. We analyzed the transcript change of *OfSerpin-3* upon the challenge of *B. bassiana* conidia using qRT-PCR methods. The mRNA level of *OfSerpin-3* significantly increased (Figure 2), also suggesting its putative role in regulating the immune response.

#### Genes involved in signal transduction

After the invasive signals from the infectious microorganisms are recognized and modulated, the signal transduction pathways will be initiated to produce the effector molecules. Four signal transduction pathways, Toll, Imd, JNK, and JAK/STAT are known to be involved in insect immunity [29]. Toll and Imd pathways are more important in sensing microbes. The Toll pathway is primarily involved in the defense against fungi and Gram-positive bacteria with lysine-type peptidoglycans (Lys-type PGNs) in their cell walls, while the Imd pathway responds to Gram-negative bacteria and some Gram-positive bacteria with meso-di-aminopimelic acid-type peptidoglycans (DAP-type PGNs) [1]. In this study, we have identified at least 46 transcripts for signal transduction from *O. furnacalis* transcriptome, which nearly included all known components in signal transduction pathways (Table 2).

**Figure 6:**
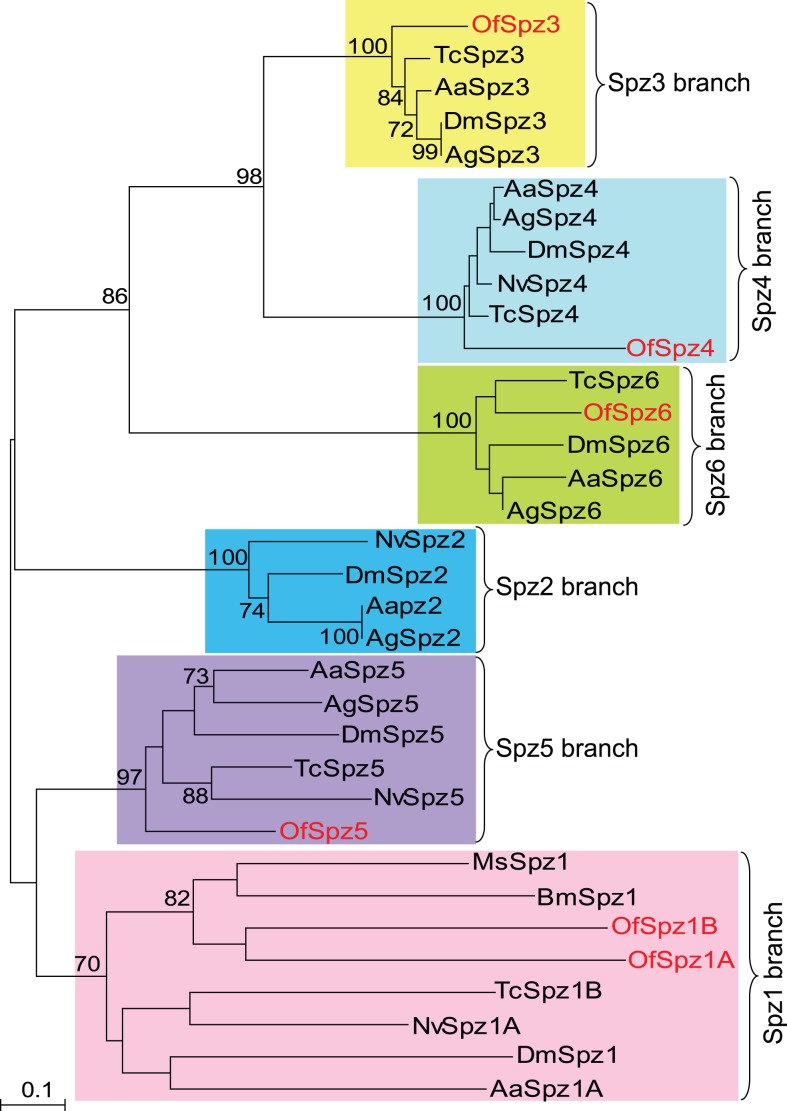
Phylogenetic analysis of the cysteine-knot domains in Spätzle from *O. furnacalis* and other insect species. The used amino acid sequences are from *Ostrinia* (Of, red), *Drosophila* (Dm), *Anopheles* (Ag), *Aedes* (Aa), *Bombyx* (Bm), *Manduca* (Ms), *Tribolium* (Tc), *Nasonia vitripennis* (Nv) cysteine-knot domains. *Ostrinia* Spzs are marked in red. The branches specific for Spz1 through Spz6 are shaded in squares. Numbers at the nodes are bootstrap values as percentage. Only bootstrap values greater than 70 are shown.

Spätzle is a ligand for the Toll receptor and activates the Toll signaling pathway [11]. *Spätzle* is present in *Anopheles* (6 genes) [27], *Drosophila* (6 genes) [26], and *Bombyx* (3 genes) [31]. *Drosophil*a Spz1, *Bombyx* Spz1, and *Manduca* Spz1 all have been demonstrated to participate in immunity [89]-[91]. ProSpätzle is consisted of an unstructured pro-domain and a C-terminal fragment that adopts a cysteine-knot structure similar to that of mammalian neurotrophins [92]. Six *Spätzle* genes have been identified from the transcriptome, with the tentative name as *OfSpz1A, 1B,* and *3-6*. Only *OfSpz1A, 1B,* and *-5* contains the complete open reading frame (Table 2). To assess the relationship between *O. furnacalis* Spätzle and other insect Spätzle proteins, we performed a phylogenetic analysis by aligning the known homologous cysteine-knot domain sequences from different insect species. The phylogenetic tree (Figure 6) suggests that all Spätzle homologs can be assigned to a 1∶1 orthologous group with one of the *Drosophila* Spätzle gene products (Spz1-Spz6). No Spätzle-2 ortholog was identified in *O. furnacalis* transcriptome. A possible reason is that Spätzle-2 gene is missing in *O. furnacalis* because of the evolutionary event. The other reason with higher possibility is that the transcript level of Spätzle-2 is low and it is not captured in the RNA-seq. As shown in Figure 6, the bootstrap value at the clade including Spz-1 is lower than in the other clades containing Spz-2 to Spz-6, indicating a lower degree of sequence conservation in Spätzle-1. It is worth noting that there are two possible *O. furnacalis* Spätzle-1 variants (*OfSpz-1A* and *OfSpz*-*1B*) in Spätzle-1 branch, which only share 33% identity in amino acid sequences. OfSpz-1A is more similar to other insect Spätzle-1, which had 56% sequence similarities with *Manduca* Spätzle-1. qRT-PCR analysis indicated that the expression of both *OfSpz-1A* and *OfSpz-1B* were induced by the *B. bassiana* conidia. It suggests that certain genes in Toll pathway are involved in innate immunity response against *B. bassiana*.

**Figure 7:**
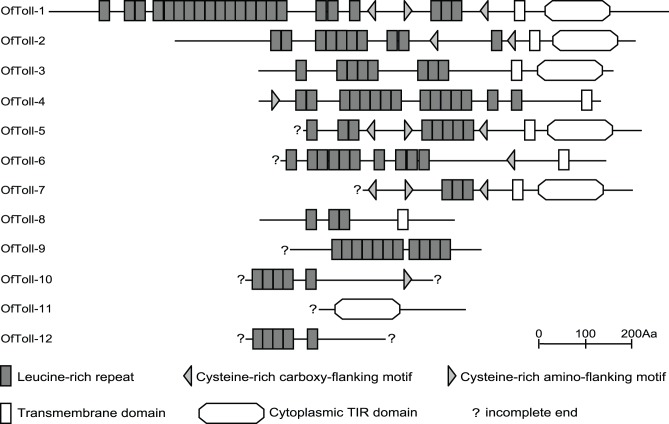
Schematic representation of the *O. furnacalis* Tolls. The domain organization was predicted using the SMART program (http://smart.embl.de/). The extracellular leucine-rich repeats are shown by small rectangles. Cysteine-rich carboxy-flanking, and amino flanking motifs are shown by left and right triangles, respectively. Putative transmembrane domains are shown by open boxes. TIR domains are represented as hexagons. Question mark means the end is incomplete.

Toll receptor plays a crucial role in insect innate immune response and acts as the signal transducer in the Toll pathway [93]. It has been identified in many insect species, including the orthoptera, hymenoptera, coleoptera, lepidoptera and diptera. Toll-like receptors (TLR) have also been detected in mammals, suggesting that Toll and TLR are evolutionarily conserved throughout the insects and mammals [94]. Insect Toll receptors and mammalian TLRs are all type I membrane proteins with an ectodomain consisting of leucine-rich repeats (LRRs), a transmembrane domain, and an intracellular Toll-interleukin homolog domain (TIR) that can transduce signals [95]. In this study, we identified 12 genes coding Toll receptors in *O. furnacalis* transcriptome datasets. These genes were designated as *OfToll1-12*. Only *OfToll1-4* and *OfToll8* genes contain the full-length encoding sequences (Table 2). Domain prediction with SMART revealed that among these 5 full-lengthed Toll-like genes, OfToll-1 through -3 is composed of the extracellular LRR, transmembrane and cytoplasmic TIR domains, while OfToll4 and OfToll8 only contains the extracellular LRRs and transmembrane domains (Figure 7). Among five incomplete *O. furnacalis* Toll-like genes with stop codon at 3′-end (*OfToll5-7*, *-9*, and *-11*), cytoplasmic TIR domain is only found in OfToll-5, OfToll-7, and OfToll-11 (Figure 7). We cannot conclude whether TIR domain is present in OfToll-10 and OfToll-12 because both 5′- and 3′-end of the cDNA sequences of these two genes are incomplete (Table 2 and Figure 7). On the other hand, the TIR domain has a more reliable determination of phylogeny than the extracellular LRR region [96]. Therefore, we were unable to conduct the convincible phylogenetic analysis for identified *O. furnacalis* Tolls. The efforts are being made to obtain the complete coding sequences for all potential *O. furnacalis Toll* genes. We investigated *Toll* gene expression after *B. bassiana* infection. Entopathogenetic fungus *B. bassiana* significantly increased the mRNA level of *Toll-1*, but decreased the transcript level of *Toll-5* (Figure 2).

In Toll signaling pathway, the cysteine-knot part of Spätzle binds to the ectodomain of the Toll receptor and thereby triggers an intracellular signaling cascade including dMyD88, Tollip, Tube, Pelle, Pellino, TRAF2, and finally results in the degradation of inhibitor protein Cactus and the nuclear import of a rel family transcription factor Dorsal/Dif [9], [97]. Contrary to the ligand-receptor diversification, components of the intracellular cascade appear to be highly conserved in insects [30]. In *O. furnacalis* transcriptome dataset, we have identified dMyD88, Tollip, Tube, Pelle, Pellino, TRAF2 with 1∶1 orthologs (Table 2). We postulated that a similar protein complex also forms in *O. furnacalis*, which will lead to the release of a Rel transcription factor (CL7451.Contig1) from a cactus-like protein (CL1201.Contig1), allowing the Rel molecular to translocate into the nucleus and activate the expression of effector genes such as antimicrobial peptides (Figure S7).

Imd pathway is also composed of multiple molecules for the signal transduction. The involved intercellular components include Imd, Fas-associated death domain protein (FADD), Dredd, IAP2, transforming growth factor β activated kinase (TAK1), Tab2, Ubc13, an inhibitor of nuclease factor κB kinase subunits β and γ (IKKβ and IKKγ), and a transcription factor Relish [98,99]. Orthologs of all these components were identified from *O. furnacalis* transcriptome (Table 2 and Figure S7). It strongly suggests that Imd-mediated immunity is conserved in Asian corn borer. Further experiments are required to verify the suggested role of each component in the immune response of *O. furnacalis*.

#### Genes for immune responsive effectors

Effector genes are induced in some specific tissues, such as fat bodies and hemocytes, following successive immune processes of signal recognition, modulation, and transduction. Part of the synthesized effector molecules are released into the hemolymph to play direct roles in phenoloxidase-dependent melanization, elimination of infectious microorganisms, apoptosis and other immune-related mechanism.

**Figure 8:**
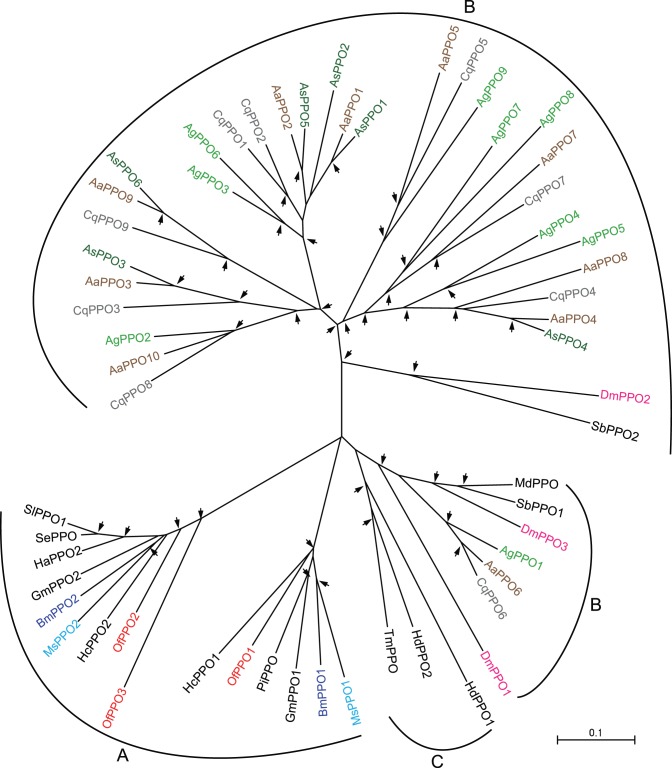
Phylogenetic analysis of prophenoloxidases (PPOs). The used amino acid sequences of 58 PPOs are from *Ostrinia* (Of), *Drosophila* (Dm), *Anopheles* (Ag), *Aedes* (Aa), *Culex* (Cq), *Bombyx* (Bm), *Manduca* (Ms), *Sarcophaga bullata* (Sb), *Musca domestica* (Md), *Armigeres subalbatus* (As), *Galleria mellonella* (Gm), *Helicoverpa armigera* (Ha), *Hyphantria cunea* (Hc), *Holotrichia diomphalia* (Hd), *Plodia interpunctella* (Pi), *Spodoptera exigua* (Se), *Spodoptera litura* (Sl), *Tenebrio molitor* (Tm). The 1∶1 and 1∶1∶1 orthologs including OfPPOs are present in the tree. A-C denotes the cluster specific for lepidopteran, dipteran, and coleopteran PPOs, respectively. For explanation of the arrows see Fig. 6.

Prophenoloxidases (PPOs) are copper-containing enzymes. They are synthesized as inactive zymogens and activated by cleavage after residue arginine at approximately residue 50 [100], [101]. A serine protease cascade contributes to the PPO activation. Active phenoloxidase (PO) catalyzed the hydroxylation of monophenols to *o*-diphenols and the oxidation of *o*-diphenols to quinones. Quinones are involved in microbial killing, melanin synthesis, sequestration of parasites or pathogens, and wound healing [7]. We have identified three PPO transcripts, and designated them as *OfPPO1*-*3* (Table 2). The total number of PPO genes in different insect species did not change significantly, except for the mosquito *Anopheles*: 3, 9, 1, 2, 2, 2 PPO genes have been identified from *Drosophila*, *Anopheles*, *Apis*, *Bombyx*, *Manduca*, and *Tribolium*, respectively [31]. Among three identified PPO fragments, only *OfPPO2* sequence was confirmed in other studies [102]. OfPPO2 is also the only one with complete coding region. Signal sequences of OfPPO1-3 are not confirmed, consistent with the situation in other insects. It suggests that PPOs are released from cells into the hemolymph by cell rupture [100]. In addition, PPO subunits form heterodimer in *Bombyx* (BomPPO1 and 2) and *Manduca* (MsPPO1 and 2), but form homodimer in *Drosophila* (DmDox-A3) [103]. We infer that *Ostrinia* PPO subunits can also form such heterodimer or homodimer. Phylogenetic analysis showed that *O. furnacalis* PPOs cluster well with other lepidopteran PPOs. The 1∶1∶1 orthologous relationship existed between OfPPO1, BmPPO1, and MsPPO1, as well as between OfPPO2, BmPPO2, and MsPPO2. It suggested that an ancestral PPO gene was duplicated after lepidoptera had diverged (Figure 8). qRT-PCR analysis of *OfPPO2* expression revealed that the mRNA level of *OfPPO2* was significantly increased after the injection of *B. bassiana* conidia (Figure 2).

Antimicrobial peptides (AMPs) are another group of immune-responsive effectors that are directly active against the infectious microorganisms. Based on their amino acid composition and antimicrobial activities, AMPs are generally classified into five groups: cecropins, insect defensins, lysozymes, proline-rich proteins, and glycine-rich proteins such as attacin [104], [105]. Analysis of the *O. furnacalis* transcriptome revealed a large number of unigenes with homology to the major families of AMPs such as lysozymes, cecropins, moricins, lebocins, gloverins, defensins, and attacins (Table 2).

**Figure 9:**
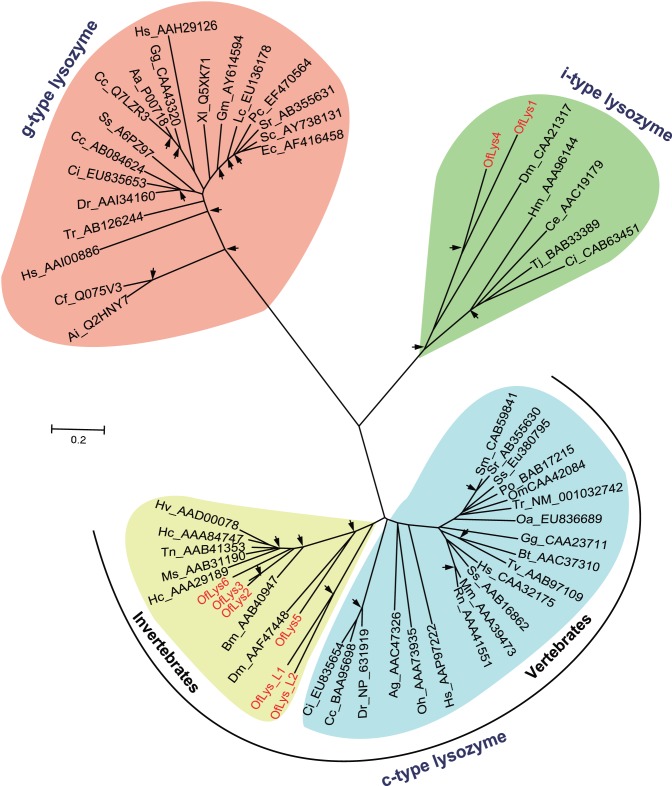
Phylogenetic analysis of lysozymes. The names of lysozyme genes used in the analysis were shown as scientific name of species followed by GenBank accession number of this specific gene. The *Ostrinia* lysozymes are marked in red. The branches specific for invertebrate c-type, vertebrate c-type, i-type, and g-type lysozymes are shaded in yellow, blue, green, and orange, respectively. For explanation of the arrows see Fig. 6.

Lysozymes catalyze the hydrolysis of the β-1,4-glycosidic bond between N-acetylglucosamine and N-acetylmuramic acids of peptidoglycans in bacterial cell walls [106]. They are divided into five major types including the c-type (chicken type), g-type (goose type), i-type (invertebrates), phage type, bacteria type, and plant type [107]. In this study, we identified six putative lysozyme genes (*OfLys1-Lys6*), and two putative lysozyme-like genes (*OfLys-L1* and *OfLys-L2*). OfLys-L1 and OfurLys-L2 have an amino acid replaced at one or both of catalytic amino acid position (glutamic acid and aspartic acid), and, therefore, may lose muramidase activity [108]. The phylogenetic analysis revealed that the c-, g- and i-type lysozymes form three independent clusters, respectively (Figure 9). Among c-type cluster, the invertebrate and vertebrate formed a subgroup respectively. OfLys1-OfLys4 is included in invertebrate c-type lysozyme subgroup. OfLys5 and OfLys6 are clustered with i-type lysozymes with a high bootstrap support. We randomly selected *OfLys2* for qRT-PCR assay. The result indicated that the expression of *OfLys2* was significantly up-regulated upon the challenge of *B. Bassiana* (Figure 2).

**Figure 10:**
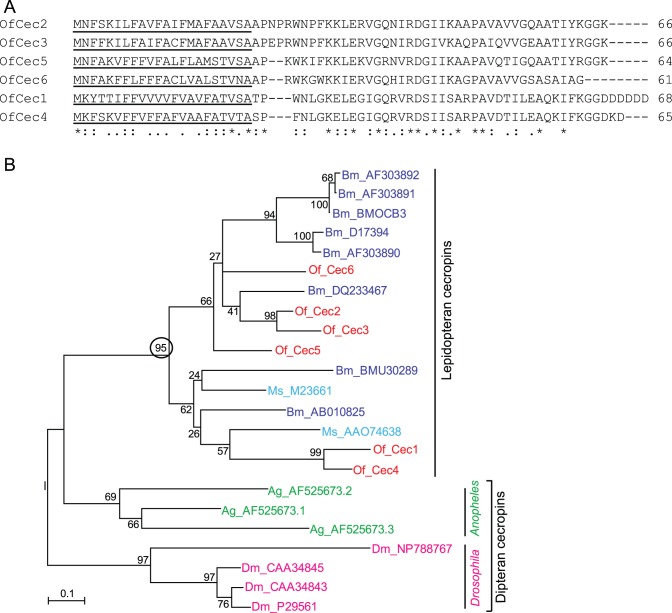
Alignment of full-length *O. furncalis* cecropins. **(A)** Completely conserved amino acids are indicated by ‘*’, conservative substitutions by ‘:’, and semiconserved substitutions by ‘.’ below the sequences. The predicted secretion signal peptide is underlined. **(B) Phylogenetic analysis of cecropins.** The amino acid sequences from 8 *Ostrinia* (Of, red), 4 *Drosophila* (Dm, pink), 3 *Anopheles* (Ag, green), 8 *Bombyx* (Bm, purple), and 2 *Manduca* (Ms, blue) cecropins were used to build the neighbor-joining tree. Numbers at the nodes are bootstrap values as percentage. Only bootstrap values greater than 70 are shown. The circled bootstrap value indicates that *Ostrinia* cecropins belong to lepidopteran cecropins.

Cecropins represent another group of linear and amphipathic peptides which adopt a-helical structure. The first cecropin was discovered in *Hyalophora cecropia* [109]. They are widely present and active against Gram-negative and Gram-positive bacteria and fungi [110]. Both *Drosophila* and *Anopheles* contain 4 *cecropin* genes and *Apis* has none [27], [29]. However, up to 13 *cecropin* genes was detected in *Bombyx* genome [31]. In this study, we have identified six putative *cecropin* genes, designated as *OfCec1*– *OfCec6* (Table 2). All identified *Ostrinia cecropin* genes contain complete coding regions, and their deduced amino acid sequences have a predicted signal peptide with 22 residues. Alignment of the conceptive protein sequences of OfCec1-6 showed that they have high similarities. For example, OfCec1 and OfCec4 share 82% identical amino acid residues, OfCec2 and OfCec3 have 85% identity in amino acid sequences (Figure 10A). Similar to the situation observed in the cecropin family in *Spodoptera exigua* [34], it is difficult to clearly distinguish that the sequence variations represent either different genes or different alleles (or alternative transcripts) from the same gene. Phylogenetic analysis showed that *Drosophila*, *Anopheles* and lepidopteran insects form cecropin gene clusters (Figure 10B). qRT-PCR assay for *OfCec3* showed that the expression level of this gene significantly increased in response to *B. bassiana* infection (Figure 2). Other than *lysozyme* and *cecropin* genes, we have identified several other unigenes encoding putative AMPs in *O. furnacalis* transcriptome dataset, including four *moricins*, three *lebocins*, two *gloverins*, one *defensin* and one *attacin* (Table 2). Expression profile analysis with qRT-PCR methods showed that all tested antimicrobial peptide genes were significantly induced in response to the *B. bassiana* injection, with the exception of *defensin* remaining unchanged (Figure 2).

In addition, oxygen-derived free radicals and gaseous radical nitric oxide are also potent immune effector molecules [111]. Therefore, genes encoding the enzymes for the conversion of reactive oxygen species (ROS) and reactive nitrogen species (RNS) are somewhat classified as immune responsive effector genes. These enzymes include peroxidase (Pox), glutathione oxidase (GTX), superoxide dismutase (SOD), catalase, thioredoxin, thioredoxin reductase, peroxiredoxin, nitric oxide synthase (NOS), NADPH oxidase (NOX) and so on [30]. We have identified some of these genes in *O. furnacalis* transcriptome, including 17 *Pox*, 6 *SOD*, 1 *catalase*, 4 *thioredoxin*, 1 *thioredoxin reductase*, 5 *peroxiredoxin*, 2 *NOS*, and 1 *NOX* (Table 2). This suggests that local production of free radicals might be also a critical component of the immune response in Asian corn borers.

## Conclusions

In summary, we sequenced and characterized the transcriptome from water-injected and *B. bassiana*-injected Asian corn borers. The transcriptome datasets obtained in this study make a significant contribution to a comprehensive sequence source for future *O. furnacalis* study, especially under the situation where its genomic information is currently unavailable. The explored immunity-related genes constitute an integrated picture of the immune network, which provides the valuable clues for a better understanding of the immune processes in *O. furnacalis* against *B. bassiana*. Immune constituent genes involved in signal recognition, modulation, transduction, and effector mechanisms have been identified and analyzed from the transcriptome. These immune repertoire genes appear to be evolutionarily conserved to different extent, and have various transcriptal profiles in response to the infection of *B. bassiana*. Functional analyses are necessary to verify our predictions. Nevertheless, the framework of information presented in this study should help clarify immune functions in an important agriculture pest and further understand the complex interaction between the insect pest and its entomopathogenic fungus.

## Supporting Information

Figure S1 **Homology analysis of** ***O. furnacalis*** **unigenes.** (A) E-value distribution. (B) Similarity distribution. (C) Species distribution. All unigenes that had BLASTX annotations within the NCBI Nr database with a cut-off E-value of 10-5 were analyzed. The first hit of each sequence was used for analysis.
(EPS)

Figure S2 **Gene ontology (GO) assignment for the** ***O. furnacalis*** **transcriptome.** GO assignments (level 2) as predicted for their involvement in (A) biological processes, (B) cellular components, and (C) molecular functions. The number of unigenes assigned to each GO term is shown behind semicolon.
(EPS)

Figure S3 **Clusters of orthologous groups (COG) classification of** ***O. furnacalis*** **unigenes.** A total of 11,462 produced functional annotations were among the 25 categories. The Y-axis shows the number of unigene in each COG term.
(EPS)

Figure S4 **Deduced amino acid sequences of 190 putative immunity-related unigenes.** The coding region sequences (CDS) were determined on the base of the BLAST results. The amino acid sequences of each unigene were deduced in the EXPASY proteomics server (http://www.expasy.org). All sequences were listed in fasta format.
(TXT)

Figure S5 **Phylogenetic analysis of catalytic domains from clip domain serine proteases (clip-SPs) and serine protease homologues (clip-SPHs).** The amino acid sequences of 16 *Ostrinia* (Of, red), 40 *Drosophila* (Dm, pink), 45 *Anopheles* (Ag, green), 13 *Bombyx* (Bm, purple), 16 *Manduca* (Ms, blue), two *Tenebrio molitor* (Tm, black), three *Holotrichia diomphalia* (Hd, black), and two *Limulus polyphemus* (Lp, black) clip-SPs and clip-SPHs were used to build the unrooted tree. A–D denotes subfamilies of insect clip-SPs. The arrows at nodes denote bootstrap value greater than 700 from 1000 trials.
(EPS)

Figure S6 **Phylogenetic relationships among serpins (Spns).** The amino acid sequences of 17 *Ostrinia* (Of, red), 6 *Drosophila* (Dm, pink), 5 *Anopheles* (Ag, green), 5 *Aedes* (Aa, brown), 28 *Bombyx* (Bm, purple), 6 *Manduca* (Ms, blue), 6 *Tribolium* (Tc, light purple); 4 *Apis mellifera* (Am, black) serpins were analyzed. The clade that groups OfSerpin-3 with other known melanization inhibitors including AgSRPN2, AaSpn2, DmSpn27A, and MsSerpin-3 was shaded in yellow. The arrows at nodes denote bootstrap value greater than 700 from 1000 trials.
(EPS)

Figure S7 **Schematic drawing of the Toll (A) and Imd (B) signaling pathway in** ***Drosophila*** **and** ***Ostrinia*** **.** Components of the putative pathway from *O. furnacalis* are predicted based on sequence similarity. The *Drosophila* gene names are followed by unigene number of their Asian corn borer orthologs (or paralogs in some cases).
(EPS)

Table S1 **Primers for qRT-PCR analysis.**
(XLS)

Table S2 **Statistics of sequencing quality.**
(DOC)

Table S3 **The ID, length, expression and functional annotation of each unigene.**
(XLSX)

Table S4 **The statistics of annotated unigenes.**
(DOC)

Table S5 **The ID, length, expression and functional annotation of each differently expressed unigene.**
(XLS)
