# Correction: Identification of Immunity-Related Genes in *Ostrinia furnacalis* against Entomopathogenic Fungi by RNA-Seq Analysis

**DOI:** 10.1371/annotation/755a38b9-ccc1-4042-baa2-1249c9da8670

**Published:** 2014-01-30

**Authors:** Yang Liu, Dongxu Shen, Fan Zhou, Guirong Wang, Chunju An

In the byline, the author order is incorrect. Please see the correct author order, order of affiliations and Citation below.

Dongxu Shen^1^, Yang Liu^2^, Fang Zhou^2^, Guirong Wang^1^, Chunju An^1^

1 Department of Entomology, College of Agriculture and Biotechnology, China Agricultural University, Beijing, China

2 State Key Laboratory for Biology of Plant Diseases and Insect Pests, Institute of Plant Protection, Chinese Academy of Agricultural Sciences, Beijing, China

Citation: Shen D, Liu Y, Zhou F, Wang G, An C (2014) Identification of Immunity-Related Genes in *Ostrinia furnacalis* against Entomopathogenic Fungi by RNA-Seq Analysis. PLoS ONE 9(1): e86436. doi:10.1371/journal.pone.0086436

