# Supplementary material for: Identification of Immunity-Related Genes in Ostrinia furnacalis against Entomopathogenic Fungi by RNA-Seq Analysis
Source: PLoS One. 2014 Jan 17;9(1):e86436. doi: 10.1371/journal.pone.0086436 (PMC3895045; doi:10.1371/journal.pone.0086436)
Supplement: Table S2 — Statistics of sequencing quality. (DOC) [file pone.0086436.s009.doc]

**Table S2. Statistics of sequencing quality**

| Sample | Total Raw Reads | Total Clean Reads | Total Clean Nucleotides (Nt) | Q20 percentage | N percentage | GC percentage |
| --- | --- | --- | --- | --- | --- | --- |
| Control | 57,411,104 | 51,594,958 | 4,643,546,220 | 96.64% | 0.00% | 49.13% |
| Treated | 57,669,432 | 52,437,534 | 4,719,378,060 | 97.04% | 0.00% | 49.16% |
