# Supplementary material for: Identification of Immunity-Related Genes in Ostrinia furnacalis against Entomopathogenic Fungi by RNA-Seq Analysis
Source: PLoS One. 2014 Jan 17;9(1):e86436. doi: 10.1371/journal.pone.0086436 (PMC3895045; doi:10.1371/journal.pone.0086436)
Supplement: Table S4 — The statistics of annotated unigenes. (DOC) [file pone.0086436.s011.doc]

**Table S4. The statistics of annotated unigenes**

| Database | Nr | Nt | Swiss-Port | KEGG | COG | GO | All annotated | Not annotated |
| --- | --- | --- | --- | --- | --- | --- | --- | --- |
| Numbers of genes | 31,277 | 18,232 | 22,455 | 20,218 | 11,462 | 13,451 | 35,700 | 26,682 |
| Percentage (%) | 50.14 | 29.23 | 36.00 | 32.41 | 18.37 | 21.56 | 57.23 | 42.77 |

(Note: A total of 62,382 unigenes were obtained in *O. funacalis* transcriptome)
